# An *in vivo* system for directed experimental evolution of *rabbit haemorrhagic disease virus*

**DOI:** 10.1371/journal.pone.0173727

**Published:** 2017-03-13

**Authors:** Robyn N. Hall, Lorenzo Capucci, Markus Matthaei, Simona Esposito, Peter J. Kerr, Michael Frese, Tanja Strive

**Affiliations:** 1 Health and Biosecurity, Commonwealth Scientific and Industrial Research Organisation, Canberra, ACT, Australia; 2 Invasive Animals Cooperative Research Centre, University of Canberra, Canberra, ACT, Australia; 3 IZSLER, Istituto Zooprofilattico Sperimentale della Lombardia e dell’Emilia Romagna “Bruno Ubertini”, Brescia, Italy; 4 School of Life and Environmental Sciences, University of Sydney, Sydney, NSW, Australia; 5 Health Research Institute, University of Canberra, Canberra, ACT, Australia; 6 Institute for Applied Ecology, University of Canberra, Canberra, ACT, Australia; University of Texas Medical Branch at Galveston, UNITED STATES

## Abstract

The calicivirus *Rabbit haemorrhagic disease virus* (RHDV) is widely used in Australia as a biocontrol agent to manage wild European rabbit (*Oryctolagus cuniculus*) populations. However, widespread herd immunity limits the effectiveness of the currently used strain, CAPM V-351. To overcome this, we developed an experimental platform for the selection and characterisation of novel RHDV strains. As RHDV does not replicate in cell culture, variant viruses were selected by serially passaging a highly virulent RHDV field isolate in immunologically naïve laboratory rabbits that were passively immunised 18–24 hours post-challenge with a neutralising monoclonal antibody. After seven passages, two amino acid substitutions in the P2 domain of the capsid protein became fixed within the virus population. Furthermore, a synonymous substitution within the coding sequence of the viral polymerase appeared and was also maintained in all subsequent passages. These findings demonstrate proof-of-concept that RHDV evolution can be experimentally manipulated to select for virus variants with altered phenotypes, in this case partial immune escape.

## Introduction

*Rabbit haemorrhagic disease virus* (RHDV) is a single-stranded, positive-sense RNA virus of the genus *Lagovirus* in the family *Caliciviridae* [1, 2]. In susceptible adult European rabbits (*Oryctolagus cuniculus*), the virus causes rabbit haemorrhagic disease (RHD), a fulminant necrotising hepatitis with a case fatality rate of approximately 90% [1]. RHDV is highly species-specific, environmentally stable and can be transmitted by direct contact, via fomites and mechanically by insects [3]. These factors, along with the high case fatality rate of RHD, prompted investigations into the use of RHDV as a biocontrol agent to manage Australia’s wild rabbits in the early 1990s [4, 5]. In 1995, the RHDV Czech strain CAPM V-351, belonging to the RHDV genogroup G2, was released in Australia and since then natural outbreaks occur regularly in wild rabbit populations, in addition to ongoing releases of the original CAPM V-351 virus. However, the effectiveness of the original strain is limited by immunity in recovered rabbits and by emerging genetic resistance in some populations [6]. Other factors impacting on the effectiveness of biocontrol with RHDV are age-dependent resistance and partial cross-protection afforded by a related but benign calicivirus (RCV-A1) that is endemic in south-eastern Australia [7].

Recent evidence has shown that wild rabbit numbers in Australia are recovering from the initial knockdown due to RHDV in the mid-1990s [8]. To facilitate long-term sustainable RHDV-mediated rabbit biocontrol in Australia, it is necessary to identify RHDV antigenic variants that are able to overcome immunity to CAPM V-351 and circulating field strains. However, even if such calicivirus strains are used in the field, over time both resistance and immunity will inevitably develop, and rabbit numbers will recover once more. It would therefore be highly desirable to have a pipeline for the continuous generation of novel virus variants that can be deployed in an ongoing fashion for enduring effective biocontrol. As no robust cell culture or reliable reverse genetics systems for RHDV have been established, any such pipeline must presently involve passaging and selecting viruses in rabbits. Here we report on the development of such an experimental *in vivo* platform for the directed evolution of RHDV variants, and demonstrate proof-of-principle that this method may be used to generate viruses with altered phenotypes.

## Methods

### Animals

All animal trials were carried out at CSIRO Black Mountain Laboratories according to the Australian Code for the Care and Use of Animals for Scientific Purposes (2013) and approved by the CSIRO Ecosystem Sciences Animal Ethics Committee (permit identifiers: CESAEC 13–01, DOMRAB).

### Animal trials

**Fig 1:**
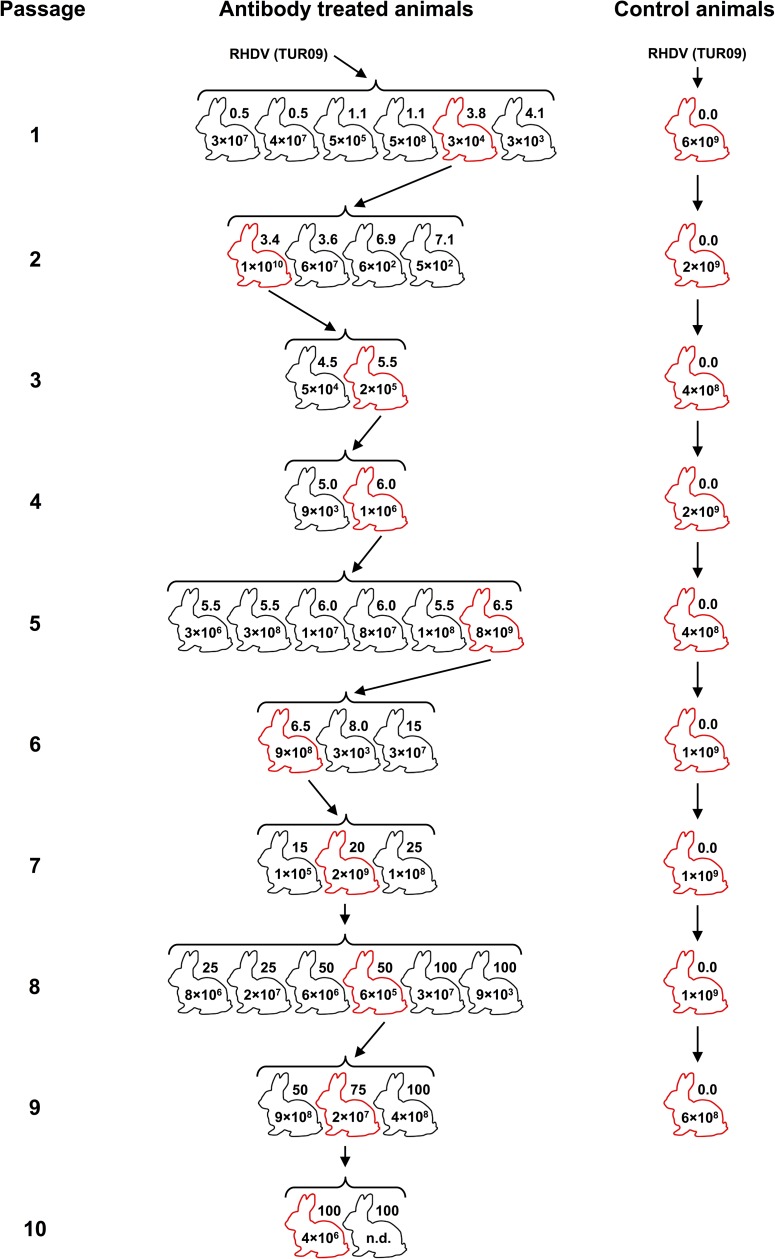
Serial passaging of RHDV in adult rabbits. Rabbits were infected orally with 1 × 10^5^−2 × 10^8^ capsid gene copies of RHDV. All rabbits within a passage received the same inoculum dose but the dose varied between passages. The RHDV-specific monoclonal antibody (mab 1H3) was administered intravenously 18−24 hours post-infection. Control animals were infected with RHDV but did not receive antibody injections. Rabbits were euthanised 3−4 days post-infection or when signs of terminal RHD were detected. A single animal from each passage (marked in red) was selected for the production of a new virus stock. Individual mab doses (μg/kg) are specified next to the rabbit outlines and virus titres in the liver, as quantified by qRT-PCR (capsid gene copies/mg liver tissue), are given inside rabbit drawings.

New Zealand White rabbits (9–14 weeks old) were housed either individually or in pairs depending on size. All rabbits were negative for RCV-A1 antibodies [9, 10]. Animals were infected orally with 0.7–1 ml of 10% clarified liver homogenate, prepared initially from a wild rabbit that died of infection with a highly virulent field strain, and subsequently from the liver of a single animal from the preceding passage. Homogenates were diluted in PBS to contain 1 × 10^5^–2 × 10^8^ RHDV capsid gene copies in the inoculum (as determined by qRT-PCR, described below). Monitoring was conducted twice daily. After 18–24 hours, rabbits were anaesthetised by intramuscular injection of either Zoletil 100 (Virbac, Peakhurst, NSW, Australia) or a combination of Xylazil-20 (Troy Laboratories, Smithfield, NSW, Australia) and Ketamav 100 (Mavlab, Logan, QLD, Australia). The RHDV-specific mouse monoclonal antibody (mab) 1H3 was diluted in sterile PBS and administered intravenously at 1–100 μg/kg. This mab was raised against the RHDV laboratory reference strain Bs89 (Genbank accession X87607) and purified to >95% purity using Protein A chromatography as described previously [11, 12]. The antibody was quantified assuming that one A280 = 1.3 mg/ml. Different doses of mab 1H3 were administered to each rabbit within a passage and the dose range was increased for subsequent passages (Fig 1). In addition to intravenous administration of the antibody, rabbits in passage 10 were given subsequent daily doses of mab 1H3 intramuscularly to maintain selection pressure. Rabbits were euthanised either three or four days post-infection, prior to the induction of strong detectable IgM responses, or when a humane endpoint was reached (whichever was earlier). Humane endpoints for all experiments were defined as 10% acute weight loss or clinical signs consistent with terminal RHD (i.e. normo- or hypothermia after pyrexia in combination with weight loss, altered mentation and/or anorexia). Euthanasia was performed by intravenous or intracardiac injection of sodium pentobarbitone (Virbac) after inducing general anaesthesia with either Zoletil 100 intramuscularly or a combination of Xylazil-20 and Ketamav 100 intramuscularly. All efforts were made to minimise suffering throughout the experimental process, e.g. by intervening when animals had fulminant RHD and by anaesthetising animals prior to intravenous injections, including those for euthanasia. No unexpected deaths occurred during these experiments, however, it must be noted that RHD is frequently peracute and infected animals may die suddenly as an expected consequence of infection. Tissue samples, including blood, bile and liver, were collected post-mortem and stored at -80°C until further processing. Liver from one animal per passage was used to prepare the inoculum for the subsequent passage until ten serial passages were completed (Fig 1).

Nine additional rabbits (one per passage from passages 2–10) were used as unselected controls to monitor the background mutation rate. These were infected with the original inoculum virus and trials were conducted as described above but without antibody administration. A liver preparation from the control rabbit in each passage was used to generate the inoculum for the control animal in the subsequent passage.

### Virus strain

A highly virulent Australian RHDV field isolate, TUR09 (Genbank accession KF594476 [13]), was used for stock virus production. This strain was selected in order to generate a genogroup G2 antigenic variant based on circulating Australian field strains. To produce an inoculum virus stock, a single adult New Zealand White rabbit was infected by subcutaneous injection with a 10% liver homogenate prepared from a wild rabbit that had died from an infection with TUR09. As expected, the laboratory rabbit died three days post-infection. Liver was harvested and a 20% tissue homogenate was prepared in PBS. The homogenate was clarified twice by centrifugation for 25 minutes at 4,700 *g*, 4°C (Allegra 6R), followed by 30 minutes at 15,000 *g*, 4°C (Beckmann Avanti J-25I). It was then filtered through a 0.8-μM syringe filter (Millex, Bayswater, VIC, Australia) followed by a 0.22-μM syringe filter (Millex) and stored at -80°C. Virus concentration (3.0 × 10^7^ capsid gene copies per μl) was determined by qRT-PCR using the RHDV-specific RHDV-RT2 forward (fw) and reverse (rv) primers as described below.

### RNA extractions

RNA was isolated from 30 mg of liver using the Qiagen RNeasy mini kit (Qiagen, Chadstone, VIC, Australia) according to the manufacturer’s instructions, after homogenisation with 1-mm glass beads (Daintree Scientific, St Helens, TAS, Australia) using a Precellys 24-dual tissue homogeniser (Bertin Technologies, Montigny-le-Bretonneux, France).

### Virus quantification

Viral load was quantified in capsid gene copy number units using the qRT-PCR method essentially as described previously [14]. Reactions were performed in duplicate using the SensiFAST SYBR No-ROX One-Step kit (Bioline, Alexandra, NSW, Australia) on a BioRad CFX96/C1000 thermal cycler platform using the primers RHDV-RT2_fw 5’-ACCCAGTACGGCACRGGCTCCCAACCAC-3′ and RHDV-RT2_rv 5’-CTATCTCCATGAAACCAGATGCAAAGGT-3′ [15].

### Virus sequencing

First-strand cDNA was synthesised from 5 μl of RNA using 500 ng of OligodT(18mer) (Geneworks, Thebarton, SA, Australia) and Invitrogen Superscript III (Life Technologies, Mulgrave, VIC, Australia) according to manufacturers’ directions.

Overlapping fragments of the RHDV genome were amplified by PCR using Platinum *Taq* DNA polymerase high fidelity (Life Technologies) using the primer sets RHDV-1 fw (5’-GTGAAARTTATGSCGGCTATGTCGCGC-3’)/RHDV-6 rv (5’-GCCATRGTYGCAAGRTTGAC AAGGTGG-3’); RHDV-7 fw (5’-GTAYTCAAGRGACCCTGTCCCCGTGG-3’)/RHDV-10 rv (5’-CATCAT CGGRGTCATGGCATACAGGCC-3’); RHDV-11 fw (5’-CACCCCATGACYATACTTGACGCCATG-3’)/RHDV-13 rv (5’-TTTTTTATAGCTTACTTTAAACTATAAACC-3’) or RHDV_end rv (5’-TTTTTTTTTTTTTTTTTTTTTTTTTTTTTATAATTTACTCTAAATTATAAACCAATTAAATTAATTAAC-3’) [13]. If necessary, the RHDV-1 fw/RHDV-6 rv fragment was split into two fragments that were amplified separately with the primer pairs RHDV-1 fw/RHDV-2 rv (5’-GCAAGTCAWAGCARCGGTCCGTTGCAC-3’) and RHDV-3 fw (5’-GAACYTDYTAGCRATCCTCATGGACTGG-3’)/RHDV-6 rv. Similarly, the RHDV-7 fw/RHDV-10 rv fragment was split into two fragments amplified with the primer pairs RHDV-7 fw/RHDV-8 rv (5’-GTTCATGAGTGAYTTGTCACTGTCTGG-3’) and RHDV-9 fw (5’-GAYGGKTGYACRCAGACCACCCACGG-3’)/RHDV-10 rv when required. Some virus genomes could be amplified in a single fragment using the primer pair RHDV-1 fw/RHDV-13 rv.

Amplicons for each virus (i.e. from each individual animal) were quantified using the Qubit dsDNA BR assay (Life Technologies) and pooled in equimolar ratios, before dilution to a final concentration of 0.14 ng/μl in UltraPure DNase/RNase-free distilled water (Life Technologies).

### DNA library preparation and Illumina sequencing

Sequencing and data analysis was performed as described previously [16], with modifications. DNA libraries were prepared from 0.7 ng of pooled amplicons using the Nextera XT DNA sample preparation kit (Illumina, Scoresby, VIC, Australia) according to manufacturer’s directions. Paired-end sequencing was performed using the Illumina MiSeq platform and a 300 cycle MiSeq reagent kit v2. Raw sequence reads have been uploaded to the NCBI Sequence Read Archive under accession number SRP096083.

### NGS data analysis and genome assembly

Sequence read quality was assessed using FastQC (http://www.bioinformatics.babraham.ac.uk/projects/fastqc/). Reads were then trimmed using Trimmomatic [17]; for each read, 15 nucleotides (nt) from the start and one nt from the end was removed, and a sliding window of four nt was applied with an average quality score threshold set to Q32. Overlapping paired-end reads were merged with FLASH [18], and reads less than 50 nt in length were discarded. Individual cleaned reads from the original inoculum virus sequence were mapped to the TUR09 genome (Genbank accession KF594476) to generate a majority consensus sequence using Geneious v8.1.6 [19]. Reads from subsequent isolates were mapped to the original inoculum consensus sequence. Sequences were labelled by passage number (P) and animal number (K), e.g. P04-K193.

For polymorphism analysis, individual cleaned reads were mapped to the majority consensus sequence for the respective isolate. Average sequence coverage was approximately 8,000. Nucleotide polymorphisms present at >1% were detected using the ‘Find variations/SNPs’ tool available through Geneious v8.1.6 [19], and called based on the difference to the original inoculum virus consensus sequence. A sequence polymorphism was defined as a nucleotide polymorphism exceeding 50% of the isolate virus population, and corresponded to a consensus sequence change for the respective isolate.

### Protein structure analysis

Amino acid variations detected by sequence analysis were mapped to the protein structures of the biological assembly of RHDV (PBD accession 3J1P) or the VP60 P domain (PDB accession 4EGT) [20], using the 3D protein structure viewer implemented in Geneious v8.1.6 [19]. The I-TASSER online server (http://zhanglab.ccmb.med.umich.edu/I-TASSER/) was used to predict three-dimensional protein structures based on amino acid sequences [21].

### Antigenic characterisation

The antigenic profiles of the RHDV reference strain Bs89 (Genbank accession X87607) [11], an antigenic variant (RHDVa) reference strain Pv97 (Genbank accession EU250330) [22], the passage 2 virus (which shared 100% amino acid identity with the parent TUR09 virus), and the variant derived by serial passaging in this study, were compared using a panel of seven lagovirus-specific mabs and a sandwich ELISA method, as previously described [11, 12, 22, 23]. Briefly, 10% liver homogenates were pre-titrated [11] and used at a dilution that gave an OD_492_ value closest to 1.2. Liver homogenates were pre-incubated with 5 μg/ml of each mab (a saturating dose of mab) for 30 minutes at 37°C. The antigen-antibody mixture was distributed to ELISA plates previously coated overnight at 4°C with hyperimmune RHDV serum diluted 1/5,000 in standard carbonate buffer. After incubation at 37°C for 1 hour, plates were washed and incubated with a mixture of the mabs 1H3, 5D11 and 2B4 at 37°C for 1 hour. Finally, horseradish peroxidase-labelled rabbit anti-mouse IgG was used to detect the binding of the mabs to the virus. For each virus, the OD_492_ value obtained with the non-binding mab 5F5 (negative control) was set as 0% inhibition. RHDV Bs89 [11] was used as the reference strain for the mabs 2B4, 2G3, 1H8, 2A10 and 1H3, while the antigenic variant RHDVa Pv97 [22] was the reference strain for the mabs 1F10 and 5D11, and their respective reactivity was set as 100% inhibition. The percent inhibition of each mab towards each virus was calculated as: the OD_492_ value of the virus tested (X) minus the OD_492_ value of the reference strain (ref), divided by the OD_492_ value of the virus tested for mab 5F5 (neg) minus the OD_492_ value of the reference strain, i.e. ((X—ref) / (neg—ref)).

## Results and discussion

We aimed to develop an *in vivo* platform for accelerated and directed evolution of RHDV antigenic variants. Since RHDV cannot presently be grown in cell culture, variant viruses were selected by serial passaging through laboratory rabbits with increasing doses of the RHDV-specific mab 1H3 (Fig 1). As a control for background substitution rates, nine virus passages were performed in animals that did not receive antibody.

### Immune selection with mab 1H3 generates highly virulent virus variants

**Fig 2:**
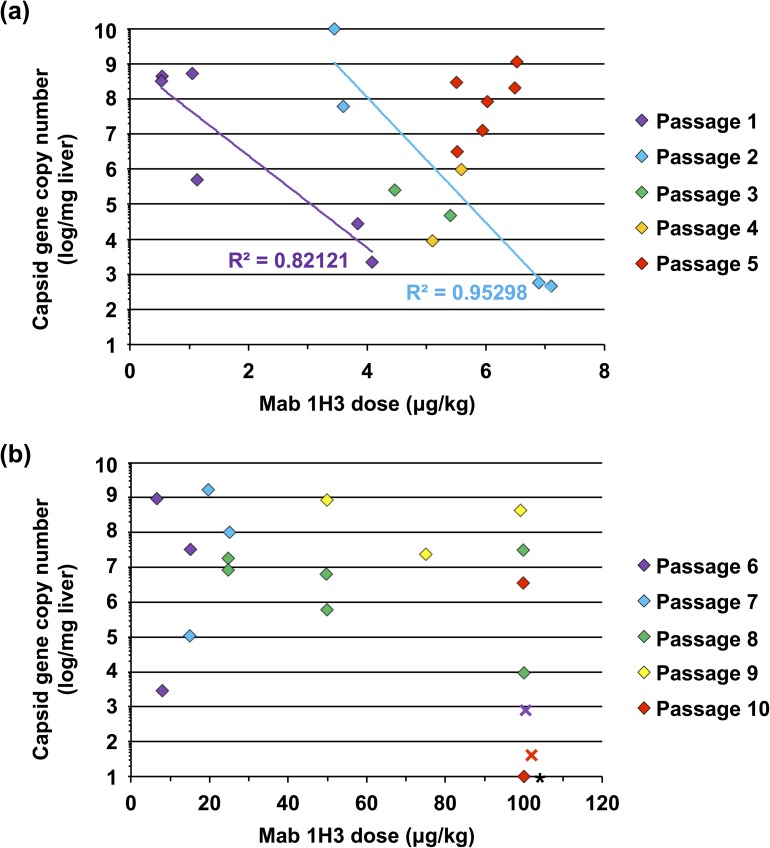
Virus load in the liver at post-mortem vs. antibody dose used for selection. Rabbits were infected orally with 1 × 10^5^–2 × 10^8^ capsid gene copies prepared from the liver of an RHDV-infected rabbit. Infection progressed for 18–24 hours before animals were passively immunised by the intravenous administration of the RHDV-specific monoclonal antibody (mab) 1H3. Animals were killed 3–4 days post-infection and virus load in the liver was quantified by qRT-PCR. **(a)** Passages 1–5; **(b)** passages 6–10. Diamonds represent virus loads from individual animals treated with mab 1H3. Virus loads from control animals ranged from 4 × 10^8^–6 × 10^9^ capsid gene copies per mg of liver (not shown). Crosses represent animals that were administered mab 1H3 at the time of infection. Virus was not detectable in the animal indicated by an asterisk. In passages 1 and 2, viral titres were clearly inversely correlated with mab 1H3 dose, as shown by the trend lines (R^2^ values shown); this correlation was not observed in subsequent passages.

Initially (i.e. in passage 1), selection of the RHDV TUR09 virus was performed using relatively low mab 1H3 concentrations (<5 μg/kg; Fig 2A). Although the virus replicated to high titres at low mab 1H3 doses (<1 μg/kg), higher antibody doses (3–5 μg/kg) restricted replication to less than 3 × 10^4^ capsid gene copies per mg of liver in passage 1, compared with 7.6 × 10^9^ capsid gene copies per mg of liver in the control animal. Interestingly, by passage 5, high-level replication was again observed for all antibody doses tested, ranging from 5.5–6.5 μg/kg (Fig 2A). This suggests that partial immune escape had occurred by passage 5, as passaged virus was able to replicate in the presence of previously inhibitory antibody concentrations. From passages 6–10, the virus replicated to high levels in the presence of mab 1H3 concentrations up to 100 μg/kg in most animals (Fig 2B). However, when high doses of mab 1H3 (i.e. 100 μg/kg) were administered concurrently with infection in passages 6 and 10 (rather than 24 hours post-infection) virus replication was suppressed, demonstrating that the virus did not completely escape antibody binding (Fig 2B). This may limit the use of the newly generated virus as a biocontrol agent, because in the wild immunity is pre-existing rather than induced. Additional experiments in naturally immune rabbits are required to address this issue. We speculate that in the initial 24-hour period post-infection, viruses from passage 6 onwards were able to reach titres high enough to overcome mab 1H3-mediated neutralisation. This suggests that the virus may have responded to the selection pressure by accelerating its replication speed, rather than via immune escape. Most importantly, however, the newly generated virus (passage 10) was still highly virulent during passaging experiments, causing terminal RHD within 4 days post-infection.

### Genetic changes in the capsid protein correlate with partial immune escape

**Fig 3:**
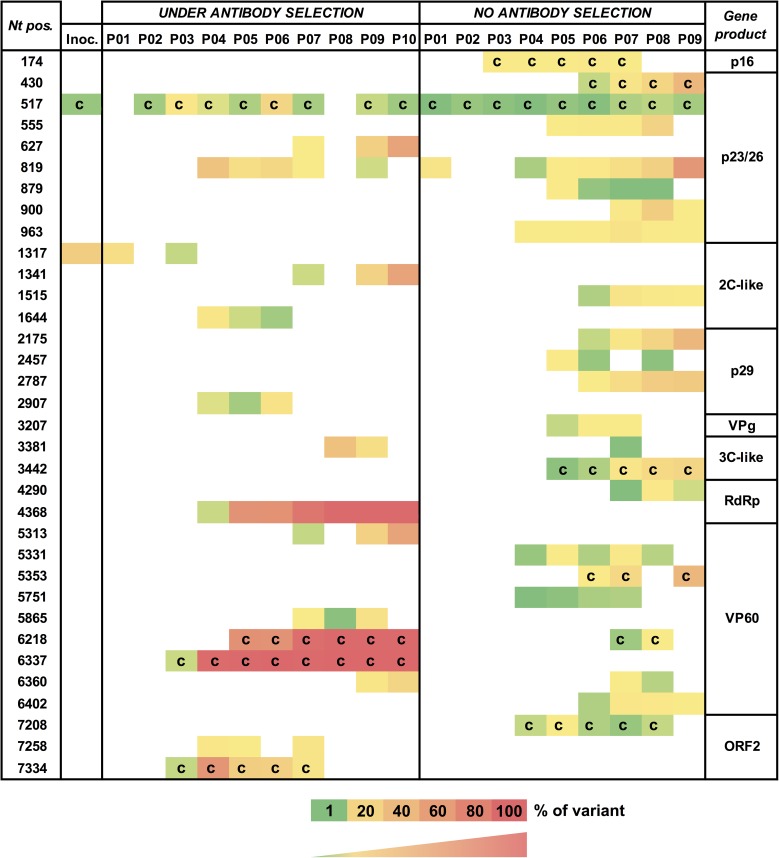
Genetic changes in serially passaged RHDV. Each row depicts a nucleotide position numbered according to the TUR09 genome (Genbank accession KF594476). Only nucleotide polymorphisms that were present at >1% and in at least three passages are shown. Each column depicts a virus isolate for the respective passage; only the isolates used for subsequent passaging are shown. ‘Inoc.’ refers to the original TUR09 inoculum (note that the inoculum contained two variable sites); ‘c’ indicates a non-synonymous (coding) substitution. The viral protein potentially affected by the polymorphism is indicated on the right. VPg = viral genome-linked protein; RdRp = RNA-dependent RNA polymerase; VP60 = major capsid protein.

The intra-host heterogeneity and majority consensus sequence of the virus population within each isolate was explored by full genome sequencing. Nucleotide polymorphisms present at >1% of the intra-host virus population were determined, in order to monitor changes over serial passages (Fig 3).

**Fig 4:**
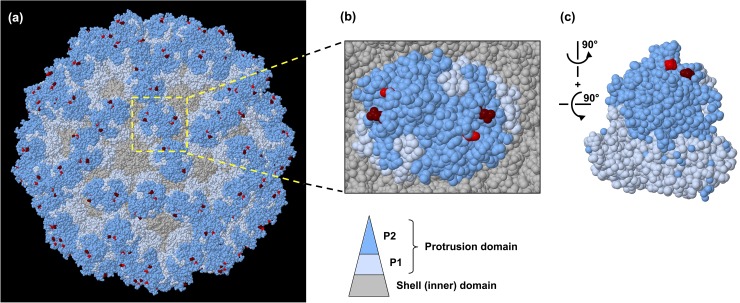
Amino acid changes in the RHDV capsid protein after serial passaging under immune pressure. **(a)** Structural model of an RHDV virion consisting of 90 VP60 dimers. **(b)** Magnified image showing the protrusion subdomains, P1 and P2, of a single VP60 dimer in the context of the entire capsid. **(c)** P domain of a single VP60 dimer. The protrusion domains P1 and P2 and the shell domain are coloured in light blue, dark blue and grey, respectively. The positions of the amino acid substitutions P305L and N345D are indicated in red and maroon, respectively.

Two consensus (>50% nucleotide polymorphism) coding mutations were detected: the first in passage 3 at position 6,337 (N345D), and the second in passage 5 at position 6,218 (P305L; Fig 3). Both substitutions rapidly became dominant and fixed in the virus population, and neither of these mutations were observed in control animal virus populations (Fig 3). The emergence of these mutations correlated with the ability of the virus to overcome increased antibody doses (Fig 2A), suggesting that these amino acid changes affected the binding of mab 1H3. The RHDV virion is composed of 180 units of the capsid protein VP60 [24]. VP60 is subdivided into the internal shell (S) and exposed protrusion (P) domain [24]. The P domain is further divided into subdomains, of which P2 is the most externally located [24]. The two amino acid mutations that appeared in passages 3 and 5 are located in the P2 domain [20], within the previously described hypervariable E (N345D) and C surface regions (P305L) [25]. When these changes were mapped to the P domain of RHDV, they were juxtaposed, suggesting that both sites may be involved in the formation of the conformational epitope for mab 1H3 binding (Fig 4). The two sites are separated by only 0.7–1.2 nm on a single VP60 monomer, implying that both amino acid changes have indeed occurred within the same epitope. Three-dimensional structural prediction using the I-TASSER server [21] did not show any evidence of major topological rearrangements due to these two amino acid changes, which in combination with the observed infectivity of the newly generated virus suggests that receptor binding was not affected (data not shown).

**Fig 5:**
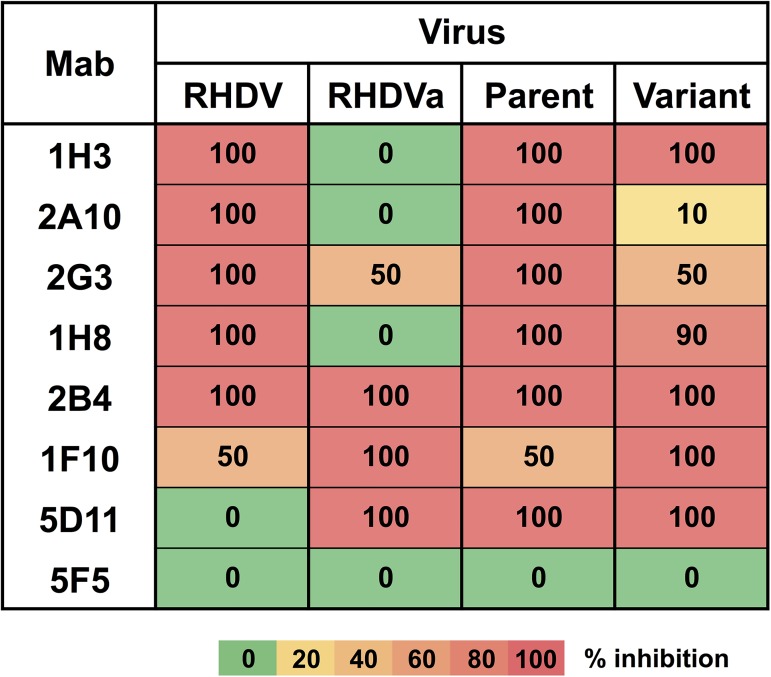
Antigenic characterisation of the variant virus by ELISA using a lagovirus-specific mab panel. Antigenic reactivity was determined using a sandwich ELISA. Mab reactivity of the original virus and the passaged virus produced in this study was compared to the laboratory reference strains RHDV Bs89 [11] for the mabs 1H3, 2A10, 2G3, 1H8 and 2B4, and the antigenic variant RHDVa Pv97 [22] for the mabs 1F10 and 5D11.

Position 305 of VP60 is located within the prominent V1-extended loop region (aa 304–314), the most exposed and diverse region on the virion surface, and adjacent to three putative histo-blood group antigen binding pockets [20]. Variation at this site has previously been reported in RHDV CAPM V-351 [26]. The V1-loop has been described as the primary determinant of virus-host interactions [13, 20]. It is also the major immunodominant epitope, and it has been shown that peptides generated from this region can stimulate the production of neutralising antibodies [20]. The homologous position in *Murine norovirus-1* is also a known antibody binding site [27]. Position 345 lies either on or adjacent to the variable loop 2 region, the second of three surface loops, however, the exact position of this region has not been well defined [20, 28]. These loops are inherently flexible, and therefore poorly structurally defined, and may thus more readily accommodate structural changes. Indeed, changes in these loops have been suggested to define the antigenicity of the calicivirus virion [20, 29]. Therefore, it is not surprising that the observed changes in response to immune selection occurred in these regions. However, in contrast to other studies that have identified single amino acid changes capable of completely abrogating antibody neutralisation, including some in caliciviruses [30–33], the two amino acid changes at positions 305 and 345 of VP60 mediated only partial escape from mab 1H3 *in vivo* and no detectable change in mab 1H3 binding *in vitro*. Antigenic characterisation by sandwich ELISA showed no change in percent inhibition over serial passage with the mab used for selection, mab 1H3 (Fig 5). However, reduced reactivity compared to the parent virus was detected with other VP60-specific mabs, i.e. mab 2A10, 2G3 and, to a lesser extent, 1H8 (Fig 5). Interestingly, increased reactivity was observed with mab 1F10, which cross-reacts more strongly with antigenic variant RHDVa viruses than with classical RHDV viruses (Fig 5). Taken together, the data confirm that the passaged virus is antigenically distinct from the parent virus and that overall, the antigenic reactivity profile of the passaged virus evolved towards an RHDVa phenotype. Although the observed amino acid changes did not affect mab 1H3 binding, the mutations caused changes in capsid conformation that clearly affected the binding of other VP60-specific mabs. Epitope maps have not been published but it was found that the epitopes for mabs 2A10, 1H3 and 2G3 are unique (L. Capucci, unpublished data). It is unknown why these amino acid changes were selected when the observed mutations did not affect mab 1H3 binding. It is conceivable that the changes affect the *in vivo* binding of mab 1H3 in a way that cannot be detected by the sandwich ELISA or alternatively, the changes could affect immune functions such as complement binding and opsonisation.

### Genetic changes in the viral polymerase under immune selection

In addition to the two coding mutations identified in VP60, there was strong evidence for selection of a synonymous C>T mutation at nucleotide position 4,368 within the RNA-dependent RNA polymerase coding region (amino acid 202 of the polymerase), affecting a codon for alanine (Fig 3). This mutation was first detected in passage 4 and rapidly became fixed in subsequent passages. This mutation was not observed in any of the unselected passages (Fig 3). The impact of this mutation on the fitness of RHDV is presently unclear but it has previously been reported that silent mutations can be selected for and enhance viral fitness through various mechanisms; for example, through codon usage bias, by improving genetic stability, and via alterations in RNA secondary or higher-order structures [34–39]. Lastly, it is equally conceivable that this silent mutation was simply a passenger mutation arising through genetic drift. Future experiments may reveal whether similar mutations will arise again under the same or similar selective pressures.

In addition to the three polymorphisms discussed above, a number of genetic changes, both synonymous and coding, were detected in the virus populations at low levels but did not become fixed. These can be presumed to have neutral or deleterious effects on viral fitness, as they were not selected for during serial passaging. Interestingly, the G27S mutation (nucleotide position 517) within the p23/26 coding region was consistently present at a relatively low frequency in animals with and without antibody treatment, in all except two passages (Fig 3). The biological significance of this polymorphism remains to be determined. Overall, the average number of polymorphisms that appeared per passage was comparable between selected and control groups (data not shown), suggesting that immune pressure did not stimulate stress-induced mutagenesis, a phenomenon that has been widely described for bacteria, eukaryotes and archaea (reviewed in [40]). However, the number of polymorphisms clearly increased in later passages compared with earlier passages in both selected and control rabbits (Fig 3). This may be due to adaptation of the virus to laboratory rabbits, since the original inoculum virus was isolated from a wild rabbit. Most importantly, there was no evidence of attenuation of RHDV over 10 serial passages *in vivo*. The passage 10 virus was highly virulent during passaging experiments, causing clinical RHD within 48 hours post-infection and the onset of terminal signs by 4 days post-infection.

In essence, this study is an experiment in adaptive evolution, monitoring genetic dynamics of the virus population over serial transfer. While neutral mutations generally take long timescales to reach fixation in a population, beneficial mutations often undergo a selective sweep and rapidly reach fixation (reviewed in [41]). This supports our observation that the two amino acid changes identified in the capsid region, along with a silent substitution in the viral polymerase, are beneficial under antibody selection (Fig 3). In contrast, none of the mutations observed in the unselected population reached fixation during the course of the experiment. Despite an initial fitness increase in passages 4 and 5, continued passaging did not produce a complete immune escape variant. This observation is consistent with the idea of ‘diminishing returns epistasis’, where the rate of fitness increase diminishes over time in the shape of a logarithmic function [42]. This pattern of fitness change is commonly seen in laboratory-evolved populations and is a process of optimisation of fitness through the gradual accumulation of beneficial mutations [42, 43]. This is in contrast to all-or-none epistasis in which a keystone mutation occurs in a background of neutral potentiating mutations leading to a sudden change in phenotype by ‘innovation’ [44]. Weaker selection is more likely to lead to a gradual fitness increase, since there are alternative pathways available for optimisation; however, keystone mutations able to overcome extreme selection pressures may not be favoured in a landscape of weaker selection pressure, and may thus be outcompeted by other less beneficial mutations through clonal interference [41]. Alternatively, strong selection pressures may favour rare, extreme and innovative mutations but may prevent the accumulation of essential potentiating mutations. Innovative mutations may also lead to a local peak in the fitness landscape, making other alternative evolutionary pathways inaccessible [45]. This may be the case for either or both of the two capsid protein changes that were observed in this study. These changes could have driven the virus into an evolutionary cul-de-sac thereby preventing further fitness optimisation.

Our findings demonstrate proof-of-principle that RHDV evolution can be experimentally manipulated to select for virus variants with altered biological characteristics. We observed two amino acid substitutions within the P2 domain of the VP60 capsid protein that rapidly became fixed in virus populations under mab 1H3 selection. Additionally, we observed selection for a synonymous substitution within the polymerase. Since high concentrations of mab 1H3 continued to inhibit the variant virus when given concurrently with infection, mechanisms other than antigenic variation may account for the fitness advantage conferred by these mutations. For example, mutants may have an accelerated replication cycle or more rapid cell entry. Taken together, the development of an *in vivo* platform for the selection of novel RHDV variants may ultimately lead to the targeted evolution of virus strains to replace current rabbit biocontrol agents.
